# Control of Sequential MTO Reactions through an MFI-Type Zeolite Membrane Contactor

**DOI:** 10.3390/membranes10020026

**Published:** 2020-02-07

**Authors:** Shusei Tanizume, Toshihiro Yoshimura, Katsunori Ishii, Mikihiro Nomura

**Affiliations:** Department of Applied Chemistry, Shibaura Institute of Technology, 3-7-5 Toyosu, Koto-ku, Tokyo 135-8548, Japan

**Keywords:** zeolite membrane, MFI-type zeolite, membrane contactor, MTO reaction, silicalite-1 coating, catalyst regeneration

## Abstract

A membrane for controlling methanol-to-olefin (MTO) reactions was developed, which featured an MFI-type zeolite membrane (Si/Al = 25) that was synthesized on a porous α-alumina substrate using a secondary growth method. Here, the H_2_/SF_6_ permeance ratios were between 150 and 450. The methanol conversion rate was 70% with 38% ethylene selectivity and 28% propylene selectivity as determined using a cross-flow membrane contactor. In order to improve the olefin selectivity of the membrane, the MFI zeolite layer (Si/Al = ∞) was coated on an MFI-type zeolite membrane (Si/Al = 25). Using this two-layered membrane system, the olefin selectivity value increased to 85%; this was 19% higher than the value obtained during the single-layer membrane system.

## 1. Introduction

Year after year, the demand for lower olefins, such as ethylene and propylene, increases owing to their popularity as raw materials for various products, including fibers, plastics, and resins. To close this gap between demand and supply, faster, more efficient production processes must be developed and implemented. To this end, methanol-to-olefin (MTO) reactions are attracting great attention as a means to quickly produce lower olefins from easily sourced hydrocarbons, such as methane or ethane [1]. The MTO reaction is a sequential process that transitions all the way from methanol through dimethyl ether (DME), olefins, paraffins, and finally aromatics via a double-cycle mechanism that was proposed by Olsbye et al. [2].

Zeolites, such as SAPO-34 (Chabazite, CHA) and ZSM-5 (MFI), are used for various commercial applications, for example, as a catalyst for ion exchange reactions, for mesopore production via alkali treatment, and for dealumination via acid treatment [3,4,5,6]. MFI-type zeolites used as catalytic membranes are aluminosilicate crystals with pore dimensions of 5.6 × 5.3 Å and are typically used for methanol-to-gasoline reactions. However, Mohammad et al. reported that the ZSM-5 catalyst in an MTO reaction was capable of 99.3% methanol conversion with 48.3% propylene selectivity over the course of 170 h under alkaline conditions [5].

Generally, a sequential reaction can be regulated by controlling the contact time between the reactants and the catalyst via the use of a membrane contactor. In cases where a fixed-bed catalytic reactor is used, it is difficult to precisely control the contact time due to diffusion processes in the environment around the catalyst. Thus, a high degree of selectivity for the intermediate products is needed in situations that require a thin catalytic membrane. Masuda et al. applied a catalytic membrane contactor using MFI-type zeolites to an MTO reaction [7] and found that the methanol conversion rate was 90% with an olefin selectivity value of 80%. Tago et al. controlled the contact time of the catalyst with a higher degree of accuracy by inactivating the reaction on the surface of the ZSM-5 membrane, resulting in 85% olefin selectivity [8]. However, the effects of a cross-flow reactor were not discussed in that study. In order to commercialize these types of membrane contactors, cross-flow-type reactors should be further developed.

Numerous studies were conducted using MFI-type zeolite membranes [7,8,9,10,11]. Sugiyama et al. developed a new silica substrate that improved gas permeation from 3.0 × 10^−6^ mol∙m^−2^∙s^−1^∙Pa^−1^ for N_2_ permeance to 108 × 10^−6^ mol∙m^−2^∙s^−1^∙Pa^−1^ for N_2_/SF_6_ permeance [9]. Ueno et al. established the amount of seed crystal coating needed to improve the selective permeation of acetic acid (AcOH). An AcOH/H_2_O separation factor of 33 with a total flux of 0.04 kg∙m^−2^∙h^−1^ was observed for the membrane coated with 5 g∙m^−2^ of seeds [10]. Zhou et al. synthesized MFI-type zeolite membranes with high ethanol selectively for the successful preparation of an extremely diluted solution (H_2_O/SiO_2_ ratio of 800) and tetra-*n*-propylammonium bromide (TPABr) as inexpensive structure-directing agents [11]. 

In this study, an MFI-type zeolite membrane contactor was developed using a cross-flow reactor. The effects of surface coating and the regeneration of the MFI-type zeolite membrane are discussed further.

## 2. Materials and Methods 

### 2.1. Preparation of the MFI-Type Zeolite Membrane

The MFI-type zeolite membrane was prepared from an α-alumina substrate (outer diameter: 10 mm, length: 30 mm) using a secondary growth method. Here, MFI-type zeolite seed crystals were prepared in accordance with the protocols from previous reports [11] and applied to the substrate via dip coating. For the synthetic gel, tetra-*n*-propylammonium bromide (TPABr, 98%; FUJIFILM Wako Pure Chemical Corporation, Osaka, Japan), tetramethoxysilane (TMOS; Shin-Etsu Chemical Co., Ltd., Tokyo, Japan), sodium hydroxide (NaOH, 97.0%, zeolites; Kanto Chemical Co., Inc., Tokyo, Japan), sodium aluminate (NaAlO_2_, Al/NaOH = 0.81; FUJIFILM Wako Pure Chemical Corporation, Osaka, Japan), and pure water were used. The composition of the resulting gel was TMOS:TPABr:NaOH:H_2_O:NaAlO_2_ = 1:0.2:0.07:200:0.04 molar ratio. The resulting synthetic gel was stirred at room temperature for 1 h, followed by hydrothermal synthesis at 180 °C for 16 h. Thereafter, it was calcined at 500 °C for 15 h.

A second layer was applied to suppress surface reactions in the membrane contactor. The composition of the synthetic gel was TMOS:TPABr:NaOH:H_2_O = 1:0.005:0.05:75. The synthetic gel was stirred for 1 h, followed by hydrothermal synthesis at 180 °C for 24 h and calcination at 500 °C for 15 h. Ammonium chloride (NH_4_Cl; FUJIFILM Wako Pure Chemical Corporation, Osaka, Japan) was used for the ion exchange process for the conversion into the H^+^ form. The membrane was then immersed in 1 M NH_4_Cl aqueous solution at 85 °C for 3 h. Thereafter, calcination occurred at 500 °C for 3 h.

### 2.2. Characterization

For the characterization of the MFI-type zeolite membrane, an X-ray diffractometer (XRD; SmartLab, Rigaku, Tokyo, Japan) and a scanning electron microscope (SEM, VE8800; Keyence, Osaka, Japan) were used. Energy-dispersive X-ray spectroscopy (EDS, JSM-7610, JEOL, Tokyo, Japan) was employed for elemental analysis. The single-gas permeances of H_2_, N_2_, and SF_6_ were measured using a bubble flowmeter at room temperature.

The gas permeance *P_i_* (mol∙m^−2^∙s^−1^∙Pa^−1^) was calculated using Equation (1), where *n_i_/t* (mol∙s^−1^) denotes the molecular permeation rate, *A* (m^2^) is, the membrane area, and Δ*p* (Pa) is the pressure difference between the membrane feed side and the permeate side. As presented in Equation (2), membrane selectivity was evaluated using the permeance ratio *α_ij_* (-).

(1) $$P_{i}\;=\frac{n_{i}}{t\;\cdot A\cdot \;\Delta p}.$$

α_ij_ = *P*_i_/*P*_j_. (2)

### 2.3. The MTO Reaction 

**Figure 1:**
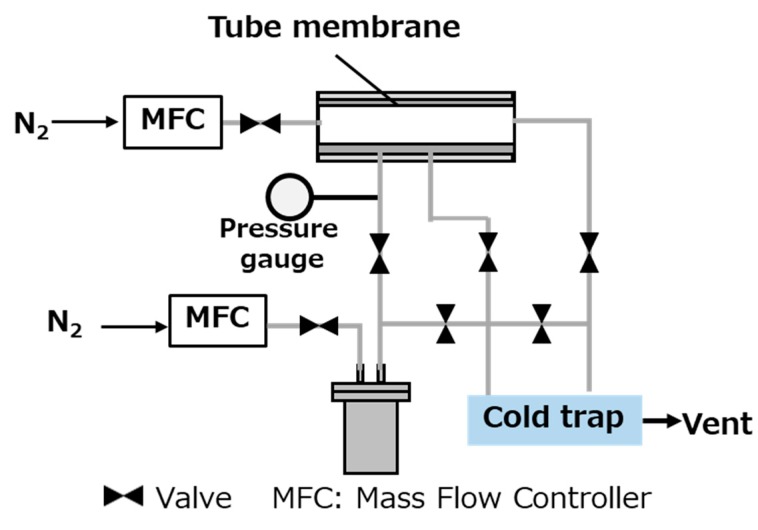
Schematic for the membrane contactor, including a diagram for the membrane module.

Figure 1 presents in detail the experimental apparatus used for the MTO reaction. Here, methanol was evaporated in the bubbler and supplied to the outside (supply side) of the membrane using N_2_ as the carrier gas. In addition, N_2_ was supplied as the sweep gas to the inside (permeate side) of the membrane to “sweep” molecules that permeated from the membrane supply side to the permeate side. The reaction was continued for 5 h at 400 °C. The carrier gas flow rate was 9.3 mL∙min^−1^, the sweep gas flow rate was 32 mL/min, and the supplied methanol vapor concentration was 0.27 mol∙L^−1^. The product composition obtained from the permeate side was analyzed using gas chromatography (GC-2014, Shimadzu, Japan). Moreover, the conversion (*X_MeOH_*) rate and the selectivity (*S_CxHy_*) were calculated using the following formulas:

(3) $$X_{MeOH}=\frac{\left(Amount\;of\;carbon\;derived\;from\;product\right)}{\left(Total\;amount\;of\;permeated\;carbon\right)},$$

(4) $$S_{C_{x}H_{y}}=\frac{\left(The\;amount\;of\;carbon\;of\;the\;target\;substance\left(C_{x}H_{y}\right)\right)}{\left(Amount\;of\;carbon\;derived\;from\;product\right)}.$$

### 2.4. Regeneration of the Catalyst via O_3_ Treatment

The catalytic property of the membrane was regenerated by heating under ozone at 100 °C for 4 h, following the protocol established in a previous study [12]. O_3_ was supplied to the outer side of the membrane at a flow rate of 200 mL∙min^−1^. 

## 3. Results and Discussion

### 3.1. Characterization

**Figure 2:**
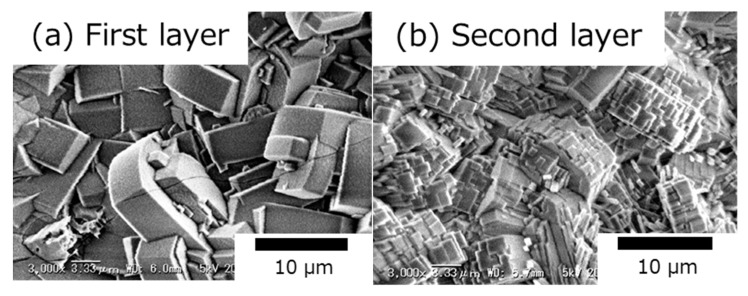
SEM images of the membrane’s surface of the (**a**) first layer and (**b**) second layer.

Figure 2 presents the SEM images of the MFI-type zeolite membrane’s surface on an α-alumina substrate. In Figure 2a, randomly oriented coffin-like crystals of about 6 to 9 µm in size can be observed (first layer), whereas, in Figure 2b, terraced structures were noted on the surface crystals of the MFI-type zeolite membrane (second layer). Even though the crystal sizes were similar on both surfaces, the crystal growth rate varied depending on the Si/Al ratio of the parent gel. Without the addition of Al to the parent gel, the prevailing reaction conditions induced much slower crystallization of the second layer. No cracks or pinholes were observed on the surface of the membrane.

**Figure 3:**
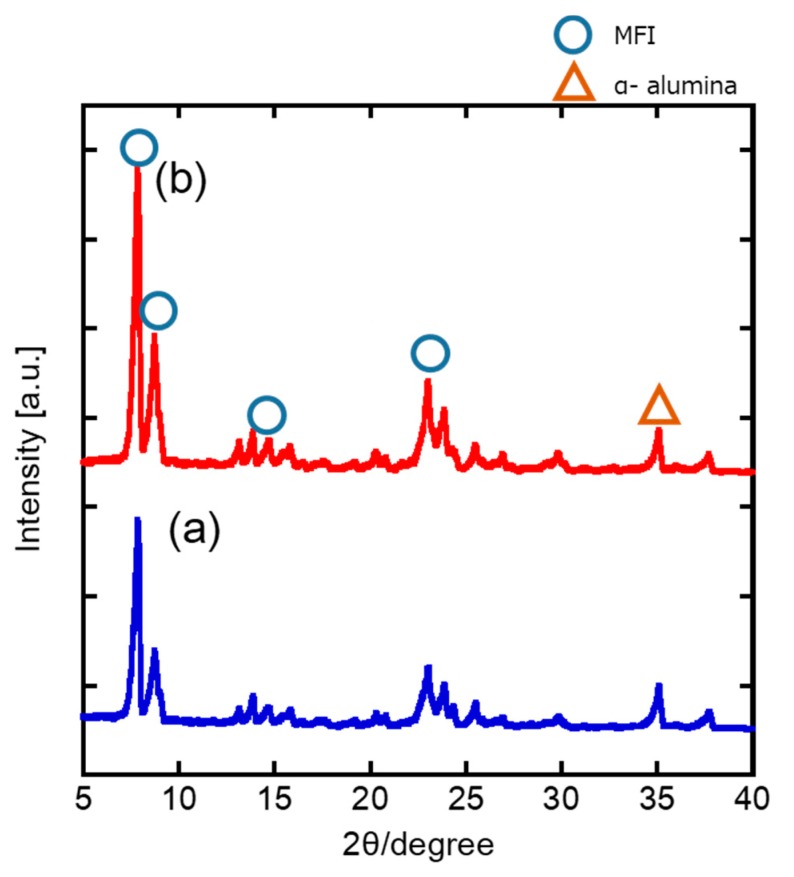
X-ray diffraction (XRD) patterns of the MFI-type zeolite membranes: (**a**) first layer; (**b**) two-layered membrane.

Figure 3 presents the XRD patterns of the prepared MFI. Here, the peaks associated with the MFI structure were observed at 7.92° (diffractive surface (011)), 8.88° (diffracted surface (020)), 14.76° (diffracted surface (031)), and 23.08° (diffracted surface (051)). The peak at around 35° was attributed to the alumina substrate. No diffraction patterns were observed, except for those linked to MFI and the alumina substrate, which indicated that pure MFI-type zeolite layers were obtained in both parent gels for the first and second layers. In addition, a more prominent peak was confirmed at around 7.9° for the second layer. The intensity ratio, which is defined as the first layer/two-layered membrane (1:1.1), was considered to be proportional to the membrane’s thickness.

**Figure 4:**
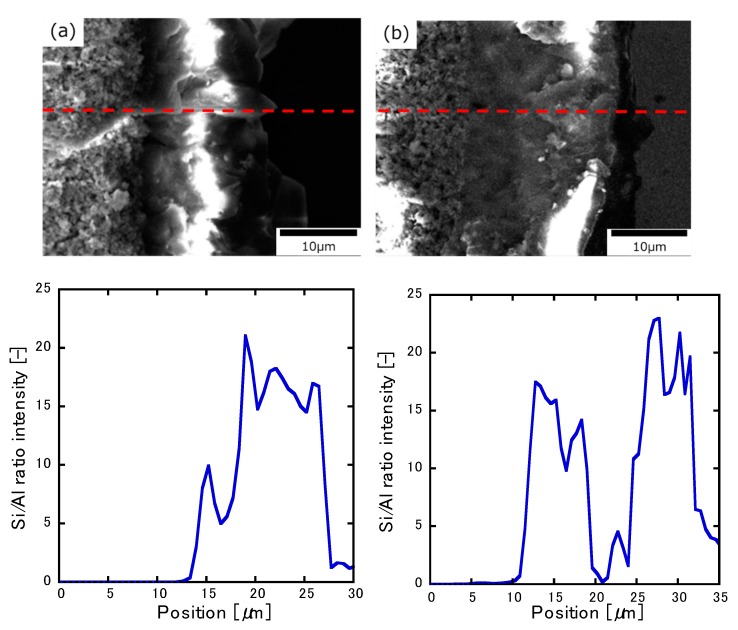
The cross-sectional view of the MFI-type zeolite membrane at (**a**) Si/Al = 25 for the single-layer membrane and (**b**) the two-layered membrane. The red lines indicate the locations where the line analysis was conducted. An average Si/Al of 0.5 μm was obtained.

Figure 4 presents the SEM image of the membrane’s cross-section and the concentration distribution of Al and Si as determined using EDS line analysis. Figure 4a presents an Si/Al ratio of 25 for the single layer, whereas Figure 4b presents the two-layered membrane with the second layer on the first layer. The thickness of the single-layer membrane (Si/Al = 25) was 10 µm. For line analysis, measurements were performed in the direction of the zeolite layer, with 0 µm being assigned to the alumina substrate. The average Si/Al ratio of the single-layer membrane was 17. Since the molar ratio of Si/Al in the parent gel was 25, it was possible that Al originating from the alumina substrate was introduced into the zeolite structure. Si existed only in the zeolite layer; thus, the membrane thickness measured from the line analysis was 8.4 µm. This was in agreement with the results of the SEM cross-section, and the same level of thickness could also be observed in the left SEM cross-sectional image. On the other hand, the thickness of the first and second layers was 8.0 and 7.2 µm, respectively, according to the EDS analysis of the two-layered membrane. The XRD patterns indicate that the MFI diffraction intensity of the two-layered membrane was smaller than that of the single-layer membrane. However, when compared with the diffraction intensity of the alumina substrate (Figure 3), the two-layered MFI-type zeolite membrane was thicker than the single-layer membrane, as denoted by the small peak observed for the two-layered membrane. This difference in the MFI diffraction intensities may be due to the crystallinity of the two-layered zeolite (Figure 2). In addition, the average Si/Al ratio of the second layer in the membrane cross-section was 18, which was slightly higher than the average Si/Al ratio of the first layer. Since the gel medium used for synthesis did not contain Al in the coating layer, Al was introduced into the MFI-type zeolite layer via the elution of the alumina substrate. However, since the average Si/Al ratio of the first layer in the membrane cross-section was 16, it can be said that the influence exerted by the Al atoms obtained from the elution of the alumina substrate was large.

### 3.2. Gas Permeation Tests

**Figure 5:**
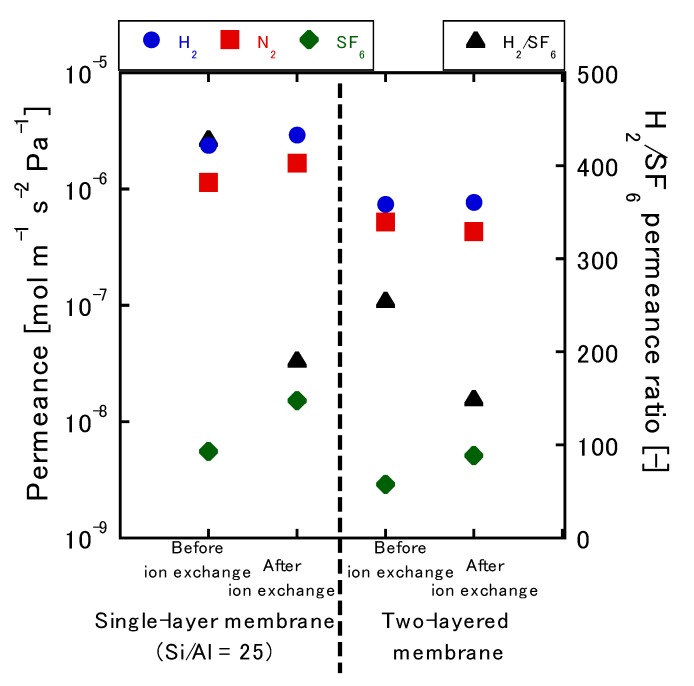
Single-gas permeance before and after ion exchange.

Next, the membrane’s ability to facilitate gas permeation was evaluated using single-gas permeation tests. Here, the gas ratios of H_2_, N_2_, and SF_6_, as well as the permeance ratio of H_2_/SF_6_, were determined (Figure 5). The gas molecular diameters were 0.29, 0.37, and 0.55 nm, respectively, and the pore diameter of MFI-type zeolite was 0.55 nm. Ideally, a membrane without cracks or pinholes should exhibit high H_2_ permeance and low SF_6_ permeance values; however, the membrane created in this study had a high H_2_/SF_6_ permeance ratio of between 150 and 450, which meant that a dense membrane without any large defects was obtained. As shown, the N_2_ permeance was 1.1 × 10^−6^ mol∙m^−2^∙s^−1^∙Pa^−1^, which was reduced to 66% when compared with the previous report [11]. The inorganic gas permeability increased as a whole after the ion exchange process, and H_2_ permeability increased 1.5-fold from 1.1 × 10^−6^ to 1.7 × 10^−6^ mol∙m^−2^∙s^−1^∙Pa^−1^. The permeability of SF_6_ increased about 5.5-fold from 5.5 × 10^−9^ to 1.5 × 10^−8^ mol∙m^−2^∙s^−1^∙Pa^−1^. This was due to the gas diffusivity of the MFI pores, which changed because the Na^+^ ions present in the MFI-type zeolite were replaced by H^+^. On the other hand, coating with silicalite-1 reduced the H_2_ permeance by almost 70% (from 2.4 × 10^−6^ to 7.5 × 10^−7^ mol∙m^−2^∙s^−1^∙Pa^−1^), whereas the SF_6_ permeance decreased by almost 50% (from 5.5 × 10^−9^ to 2.9 × 10^−9^ mol∙m^−2^∙s^−1^∙Pa^−1^). Relative to the size of the molecule, the extent to which the H_2_ permeance decreased was quite large. This could be explained by the increase in the total thickness of the membrane due to the coating of the second layer. In the ion exchange process using the two-layered membrane, the H_2_ permeance changed from 7.4 × 10^−7^ to 7.6 × 10^−7^ mol∙m^−2^∙s^−1^∙Pa^−1^, representing a relatively small difference. On the other hand, the permeability of SF_6_ increased 1.75-fold from 2.9 × 10^−9^ to 5.1 × 10^−9^ mol∙m^−2^∙s^−1^∙Pa^−1^. From this, the same trend as seen in the Si/Al = 25 single-layer membrane was observed.

### 3.3. MTO Reaction

**Figure 6:**
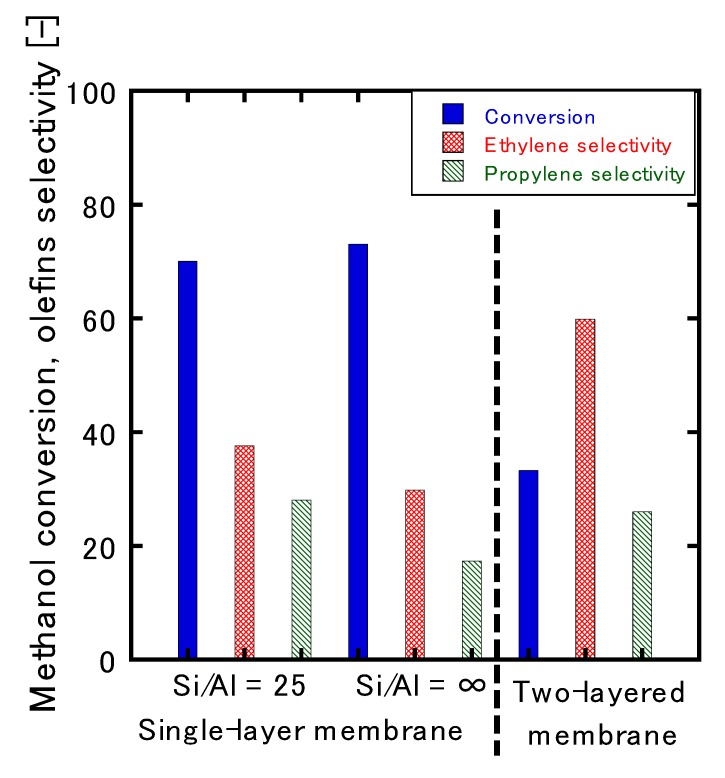
Effect of the Si/Al ratio and the single- and two-layered membranes on the conversion rate and olefin selectivity.

Figure 6 presents the initial conversion and selectivity of the MTO reaction tests for the single-layer and two-layered membranes. The Si/Al ratios of the parent gel for the single-layer membrane were 25 and ∞. Here, the value obtained 15 min after the start of the reaction was defined as the initial conversion rate/selectivity.

When the ZSM-5 single-layer membrane was used, the conversion rate was 70%, the ethylene selectivity was 38%, and the propylene selectivity was 28%. On the other hand, conversion on the two-layered membrane was 33%, which was more than half of that observed for the ZSM-5 single-layer membrane without any coating; however, the ethylene and propylene selectivity values were 60% and 25%, respectively, and the overall selectivity for olefin was 85%. It was theorized that this trend was due to the suppression of the reaction on the membrane’s surface by the second layer. Therefore, it appeared that permeation of the membrane could be controlled by using the appropriate starting material. Figure 4 shows that Al was also introduced into the zeolite of the second layer; the reaction was expected to proceed without any notable influence on the selectivity. In this study, the reaction on the membrane’s surface could be suppressed because the extent of crystallinity was insufficient (Figure 2), and there was the possibility that the Al detected by EDS was not contained within the zeolite framework. In addition, it can be observed from Figure 4 that the Si/Al intensity between 20 and 25 μm was lower than that observed at another position because the concentration of the alkali during the synthesis of the second layer was high. However, it was not clear how this interface influenced the reaction. In a previous report [8], 90% olefin selectivity was obtained by suppressing the reaction to paraffin and aromatic compounds that proceeded on the membrane’s surface; thus, the reaction in this study could be similarly suppressed. Table 1 presents the O/P ratios of the ZSM-5 single-layer and two-layered membranes. Here, the ratio of olefins to paraffins (hereafter referred to as the O/P ratio) of the two-layered membrane increased from 13.1 to 26.6; this was further evidence that reaction suppression occurred.

Even though MTO reaction tests were conducted in this study using two types of membranes with different Si/Al ratios, there was not much difference in the reactivity in relation to the Al content. With Si/Al = 25, 70% olefin conversion, 38% ethylene selectivity, and 28% propylene selectivity were obtained, whereas 73% olefin conversion, 28% ethylene selectivity, and 15% propylene selectivity were obtained when Si/Al = ∞. The reason for the conversion and olefin selectivity observed when Si/Al = ∞ could be that the Al concentration in the MFI-type zeolite layer remained at the same level due to the elution of Al from the alumina substrate (Figure 4). In addition, the deactivation of the catalyst was very fast, regardless of the amount of Al; within 60 min of the start of the reaction, reactivity dropped sharply from an olefin conversion rate of 70% to 59%, and selectivity dropped from 65% to 7%. Most notable was the fact that, after the reaction, the membrane was entirely black due to the deactivation of the catalyst via coke deposition.

In addition, both the conversion rate and the selectivity were about 20% lower than the values reported in the previous studies; this was attributed to differences in the test methods. In this study, the reaction was carried out via the cross-flow method, whereas the dead-end method was used in previous studies [7]. In the cross-flow method, MeOH molecules permeated the membrane due to a partial pressure difference, and all the MeOH molecules that did not permeate the membrane flowed to the vent side. On the other hand, MeOH molecules in the dead-end method permeated the membrane via a pressure difference between the outside and inside of the membrane, and all the MeOH molecules that were outside of the membrane also permeated the inside of the membrane. In the current study, the vapor pressure of MeOH was 28 kPa, whereas the previous study reported a value of 0.67 kPa, which was 1/20 of the value observed in the current study. The reaction times for both methodologies are notably different. In the dead-end method, the pressure was low because the reactants tended to stay within the zeolite layer, thereby extending their contact time with the catalyst. On the other hand, the pressure tended to be high in the cross-flow method because permeation of the zeolite layer was a fast process, and the MeOH molecules did not readily react. Thus, while a higher conversion rate was obtained in the previously reported experiment using the dead-end method, the cross-flow method had the advantage of being more selective toward the generation of olefin. In Table 1, the ratios of products derived from the carbon number of olefins and paraffins (the O/P ratio) are summarized. Generally, the values observed in the previous studies approximated the results observed on the membrane permeation side. When comparing the two, an O/P ratio of 13.1 was obtained in the current study, which was 3.5-fold greater than that (an O/P ratio of 3.5) observed in the previous report. This was clear evidence that the cross-flow method was preferentially selective toward the generation of olefin.

### 3.4. Catalyst Regeneration

**Figure 7:**
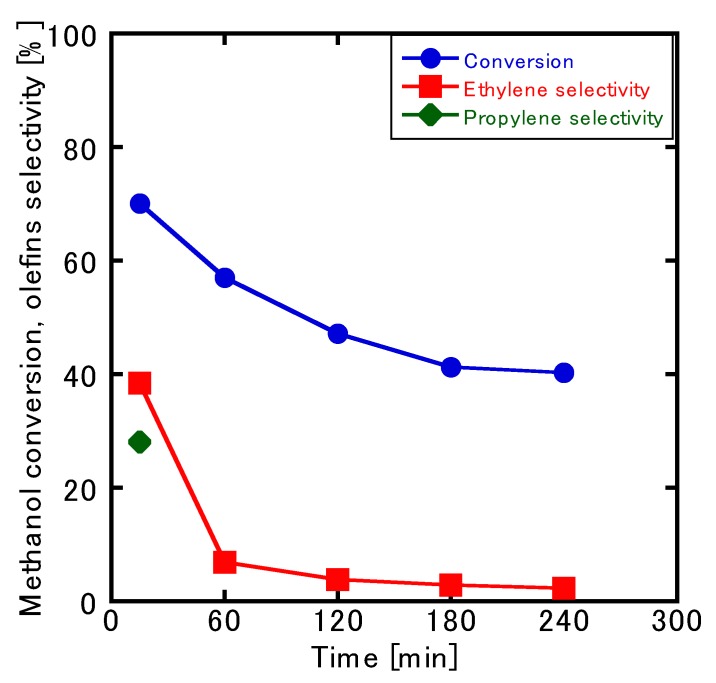
Changes in the conversion and selectivity over the course of the MTO reaction in an Si/Al = 25 membrane.

**Figure 8:**
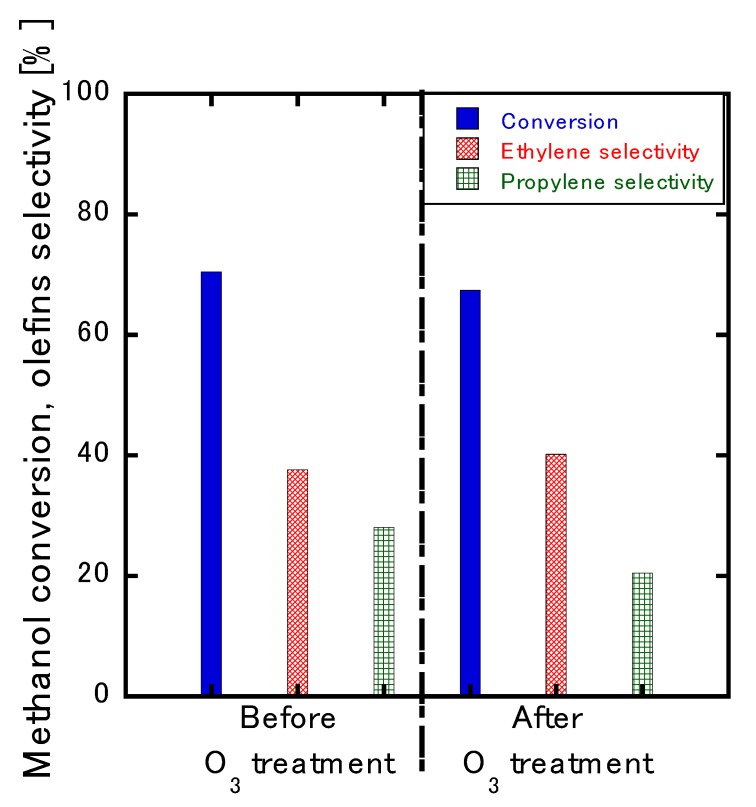
Comparison of the initial conversion rate and selectivity before and after O_3_ treatment.

Figure 7 presents a plot of the course of the reaction’s time against the conversion and selectivity of the MTO reaction in the ZSM-5-type single-layer membrane. The conversion rate and selectivity both peaked at 15 min and then experienced a rapid decrease. Almost no olefin was obtained 240 min after the start of the reaction, and the ethylene selectivity was only 4%, which resulted in a large amount of DME. Propylene was not observed after 15 min, which indicated that the catalyst was deactivated by the formation of coke on the membrane surface. This deactivation issue was, however, resolved upon treatment with O_3_ (Figure 8).

The ozone-treated membrane exhibited 67% methanol conversion, 40% ethylene selectivity, and 25% propylene selectivity. When the results before and after ozone treatment were compared, the conversion rate and selectivity decreased slightly by about 2% to 5%. This was the result of the removal of coke via oxidation into carbon dioxide. In a previous report [12], the membrane was blackened before the O_3_ treatment was performed; however, the film turned white upon treatment with O_3_. In addition, the MFI-type zeolite crystal was not destroyed during the treatment process because the film’s performance and the XRD patterns exhibited no deterioration after O_3_ treatment.

**Figure 9:**
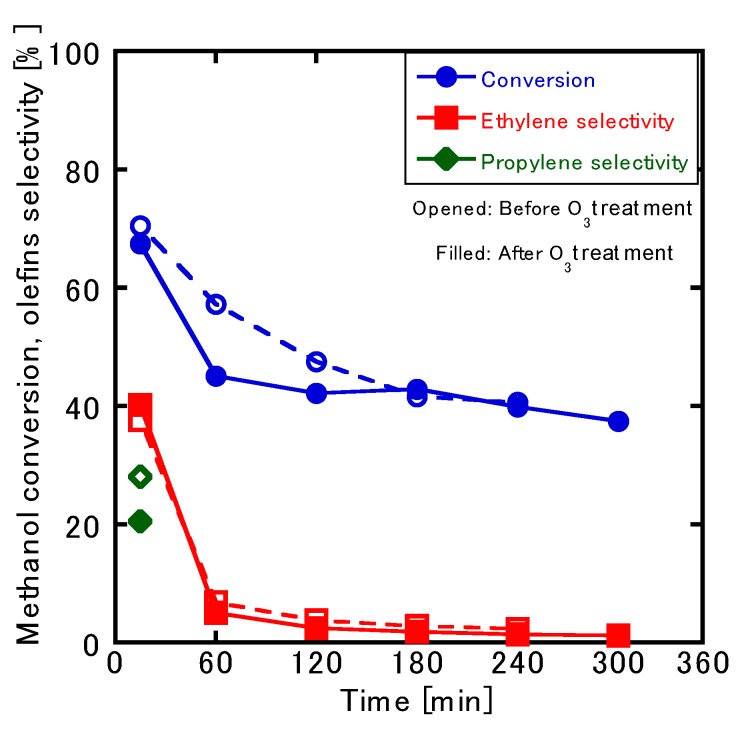
Comparison of the conversion and selectivity rates before and after O_3_ treatment.

Figure 9 presents a plot of the changes in the conversion rate and reaction selectivity against time before and after O_3_ treatment. Here, it can be observed that, although the catalyst’s performance was temporarily recovered by the O_3_ treatment process, both the conversion rate and the selectivity decreased just as rapidly as before O_3_ treatment. In particular, the rate of reduction for the conversion value observed at the 60-min mark was large; about 57% was obtained before O_3_ treatment, but only about 45% was noted after treatment. It is possible that the regeneration of the catalyst by O_3_ treatment was inadequate; thus, finding more effective means of prolonging the catalyst’s performance should be a principal goal for the future development of contactor-type membrane reactors.

## 4. Conclusions

An MFI-type zeolite membrane contactor that was synthesized using the cross-flow method was shown to be effective for MTO reactions. Here, methanol conversion was 70% with 38% ethylene selectivity and 28% propylene selectivity when the MFI-type zeolite membrane had an Si/Al ratio of 25. By applying the coating of the MFI -type zeolite layer without Al, the ethylene selectivity and propylene selectivity were improved to 60% and 25%, respectively, and the total olefin selectivity also increased to 85%. This was attributed to the suppression of the surface reaction by the MFI-type zeolite layer coating. The catalytic activity for the MFI-type zeolite membrane contactor could be regenerated by treatment with O_3_.

## Figures and Tables

**Table 1:** Comparison of the olefin:paraffin ratio (O/P ratio) of the products.

Name	Experimental Method	O/P Ratio (-)
Single-layer membrane (Si/Al = 25)	Cross-flow	13.1
Previous study [7]	Dead-end	3.5
Two-layered membrane	Cross-flow	26.6
